# Cardiac Regeneration from Activated Epicardium

**DOI:** 10.1371/journal.pone.0044692

**Published:** 2012-09-20

**Authors:** Bram van Wijk, Quinn D. Gunst, Antoon F. M. Moorman, Maurice J. B. van den Hoff

**Affiliations:** Heart Failure Research Center, Academic Medical Center, Amsterdam, The Netherlands; Northwestern University Feinberg School of Medicine, United States of America

## Abstract

In contrast to lower vertebrates, the mammalian heart has a very limited regenerative capacity. Cardiomyocytes, lost after ischemia, are replaced by fibroblasts. Although the human heart is able to form new cardiomyocytes throughout its lifespan, the efficiency of this phenomenon is not enough to substitute sufficient myocardial mass after an infarction. In contrast, zebrafish hearts regenerate through epicardial activation and initiation of myocardial proliferation. With this study we obtain insights into the activation and cellular contribution of the mammalian epicardium in response to ischemia. In a mouse myocardial infarction model we analyzed the spatio-temporal changes in expression of embryonic epicardial, EMT, and stem cell markers and the contribution of cells of the Wt1-lineage to the infarcted area. Though the integrity of the epicardial layer overlaying the infarct is lost immediately after the induction of the ischemia, it was found to be regenerated at three days post infarction. In this regenerated epicardium, the embryonic gene program is transiently re-expressed as well as proliferation. Concomitant with this activation, Wt1-lineage positive subepicardial mesenchyme is formed until two weeks post-infarction. These mesenchymal cells replace the cardiomyocytes lost due to the ischemia and contribute to the fibroblast population, myofibroblasts and coronary endothelium in the infarct, and later also to the cardiomyocyte population. We show that in mice, as in lower vertebrates, an endogenous, epicardium-dependent regenerative response to injury is induced. Although this regenerative response leads to the formation of new cardiomyocytes, their number is insufficient in mice but sufficient in lower vertebrates to replace lost cardiomyocytes. These molecular and cellular analyses provide basic knowledge essential for investigations on the regeneration of the mammalian heart aiming at epicardium-derived cells.

## Introduction

Cardiac disease leads to the highest levels of morbidity and mortality worldwide. Due to their limited regenerative capacity, cardiomyocytes lost as a result of ischemic damage are replaced by non-contractile fibrotic cells rather than by new cardiomyocytes [1], [2]. It has long been recognized that a potential cure, infers replenishment of cardiomyocytes from exogenous or endogenous sources. Application of stem cells has gained a lot of interest but the outcome is, thus far, rather disappointing; homing, survival and integration of newly formed cardiomyocytes are serious hurdles in regenerative cardiac medicine [3], [4]. Stimulation of the endogenous regenerative capacity seems an attractive alternative for the stem cell approach [5], [6]. In adult zebrafish, cardiac regeneration is found upon amputation of the cardiac apex, whereas in the mammalian heart such regeneration is limited to the first week after birth [7]. During this regenerative process the epicardium was found to play a crucial role [8]–[10]. Also from a developmental stance the epicardium is an interesting source, as (1) the epicardium is derived from a progenitor pool that also provides cardiomyocytes to the inflow of the heart [11], (2) epicardium-derived cells are essential regulators of cardiac growth [12] and (3) the epicardium contributes cells to the coronaries and interstitium [13]. Although it has been reported that embryonic epicardial genes are re-expressed in response to cardiac injury, little is known of the role of the epicardium in homeostasis and regeneration of the adult mammalian heart [14]–[17]. Recently, it has been shown that upon a myocardial infarction a limited number of epicardium-derived cells have the capacity to contribute cardiomyocytes to the infarct [18], offering a novel inroad to cardiac regeneration.

A prerequisite to developing regenerative strategies involving the epicardium is to understand the response of the epicardium to myocardial injury. To investigate the role of the epicardium in endogenous regeneration, we induced a myocardial infarction (MI) in mice in which the Wilm's Tumor 1 (Wt1)-lineage is genetically labeled. At various time points post-MI the cellular and molecular responses of the epicardium were analyzed. We observed that epicardial cells overlaying the ischemic area had disappeared one day post-MI. The epicardium bordering the ischemic area (border zone) was found to transiently re-express embryonic epicardial markers genes (Wt1, Tbx18, Raldh) and to initiate proliferation. At three days post-MI a new layer of epicardium with extended extracellular matrix had formed over the infarcted area expressing embryonic epicardial marker genes. In this epicardium, genes important for Epithelial-to-Mesenchymal-Transition (EMT) were expressed (Snai1, αSMA) and mesenchyme was observed to populate the subepicardial space. Analysis of the Wt1-lineage showed that the newly-formed epicardium and a large portion of the mesenchyme populating the subepicardial space, belonged to this lineage. Whereas most of these cells populated the infarcted area replacing the killed cardiomyocytes, immunofluorescent analysis showed that a small portion differentiated into myofibroblasts, endothelial cells, and cardiomyocytes.

## Materials and Methods

### Animals

All experimental procedures complied with national and institutional guidelines. The Animal Welfare Committee (DEC) of the University of Amsterdam approved this study and registered it as DAE101301 “Analyse van de epicard-afkomstige cellen in de regeneratie respons na een hartinfarct”. The Wt1^Cre^ transgenic line contains an IRES-EGFP-Cre cassette 17 bp downstream of the translation stop site of the Wt1 gene in the BAC clone RP23-266M16 (http://bacpac.chori.org). This BAC clone ranges from −127 kbp to +11.5 kbp relative to the transcription start site of Wt1 [19]. The Wt1^Cre^ mice were bred with the R26R line [20] to visualize the Wt1 lineage. All mice were bred on a FVB background (Harlan, Indianapolis, IN) and wild-type littermates served as controls.

### Surgical induction of a myocardial infarction

A myocardial infarction (MI) was induced by ligation of the left anterior descending (LAD) coronary artery at 8–12 weeks of age (20 gram body weight) as previously described [21]. Briefly, mice were anesthetized through intraperitoneal injection of Fentanyl (0.07 mg/kg), Dormicum (7 mg/kg), Dex-Domitor (0.35 mg/kg) and atropine (0.05 mg/kg). The mice were orally intubated using a 20-gauge intravenous catheter, mechanically ventilated (MiniVent, Harvard Biosciences), and placed on a heating pad to maintain body temperature. A left thoracotomy was performed at the third intercostal space, where muscles were dissected. The LAD was permanently ligated using a 9-0 unabsorbable ethilon suture. After visual verification of anemia and akinesis of the apex and anterior-lateral wall to ensure coronary occlusion, the thorax was closed in layers. The anesthesia was antagonized using Antisedan (2.5 mg/kg) and Flumazenil (0.5 mg/kg). After detubation, mice were kept warm until fully recovered. At least three mice were sacrificed at 1, 2, 3 days, 1 and 2 weeks, and 1 and 3 months after MI. Four hours before sacrifice the mice were injected intraperitoneally with 50 mg/kg 5′-Bromo-2′-Deoxyuridine (BrdU; Sigma) solution. After sacrifice the hearts were flushed with phosphate-buffered saline (PBS) and cleaved. One half was snap frozen in liquid nitrogen and the other half was fixed overnight in freshly dissolved 4%(w/v) paraformaldehyde (PFA) in PBS. The fixed hearts were processed, embedded in paraplast and sectioned at 8 µm for in situ hybridization and immunofluorescent analysis.

### Quantitative PCR analysis

The frozen hearts were mounted and 20 µm thick sections were cut using a cryotome. Twenty sections were collected in an eppendorf tube and an additional one was mounted for histological staining to evaluate whether the sections contained an extensive part of the infarct. If no or limited infarcted tissue was found the sample was excluded from the analysis. Total RNA was isolated from the sections using the RNeasy mini kit (Qiagen) according to the protocol of the supplier. One mg total RNA was converted into cDNA using a anchored polyT primer and SuperScript II Reverse Transcriptase (Invitrogen) in a 20 µl reaction. The RT reaction was diluted to 100 µl. Two µl single strand cDNA was used in each 5 µl qPCR reaction containing 10 pMol of the respective forward en reverse primer and LightCycler 480 SYBR Green I Master. The following primers were used: Gapdh-FW: TGTCAGCAATGCATCCTGCA, Gapdh-RV: CCGTTCAGCTCTGGGATGA, Wt1-FW: CAGATGAACCTAGGAGCTACCTTAAA, Wt1-RV: TGCCCTTCTGTCCATTTCA, Tbx18-FW: GTGGAGTCATACGCATTCTGGA, Tbx18-RV: GTGAGGATGTGTAGCAGGGACA, Snai1-FW: CTTGTGTCTGCACGACCTGT, Snai1-RV: CAGGAGAATGGCTTCTCACC, Snai2-FW: TCGTCGGCAGCTCCACTCCA, Snai2-RV: TGTCAGAGGAAGGCGGGGGAC, Raldh1-FW: TCGAGTCCCTGCCCCACTGG, Raldh1-RV: TCCACGTGGCAGATGACCTCCT, Raldh2-FW: ATGAGTGGCACGACGCCGTC, and Raldh2-RV: GCTGGAAGGCTGCACGAGCA. After an initial incubation of 5 min 95°C, the cDNA was amplified in 40 cycles of 10 sec 95°C, 20 sec 60°C, and 20 sec 72°C using a Roche Lightcycler in 384 plates. The starting concentration in each reaction was calculated using the program LinRegPCR version 12.9 [22]. The observed value of Gapdh was used to correct each sample for differences in input.

### In situ hybridization

In situ hybridization was performed essentially as previously described. [23] Sections were deparaffinized, rehydrated in a graded ethanol series and incubated with 10 mg/ml proteinase K dissolved in PBS for 15 min at 37°C. The proteinase K activity was blocked by rinsing the sections in 0.2% glycine in PBST (PBS+0.05%Tween-20) for 5 min. After rinsing in PBS, the sections were postfixed for 10 min in 4% PFA and 0.2% glutaraldehyde in PBS, followed by rinsing in PBS. After prehybridization for at least 1 hr at 70°C in hybridization mix (50%formamide, 5×SSC (20×SSC: 3 M NaCl, 0.3 M tri-sodium citrate, pH4.5), 1% blocking solution (Roche), 5 mM EDTA, 0.1% 3-[(3-Cholamidopropyl) dimethylammonio]-1-propanesulfonate (Sigma; Steinheim, Germany), 0.5 mg/ml heparin (BD Biosciences; Erembodegem, Belgium), and 1 mg/ml yeast total RNA (Roche), a digoxigenin (DIG)-labeled probe was added to the hybridization mix in a final concentration of 1 ng/ml. Probes specific to cardiac Troponin I (cTnI), Raldh1, Raldh2, Wt1, Tbx18, Snai1, Periostin and Fstl1 were used. After overnight hybridization, the sections were rinsed with 2×SSC, followed by two washes with 50% formamide, 2×SSC, pH 4.5, at 65°C, and rinsing in TNT (0.1 M Tris-HCl, pH7.5, 0.15 M NaCl, 0.05% Tween-20) at room temperature. Subsequently, the sections were incubated for 1 hr in MABT-block (100 mM Maleic Acid, 150 mM NaCl, pH7.4, 0.05% Tween-20, 2% blocking solution), followed by 2 hours incubation in MABT-block containing 100 mU/ml alkaline phosphatase-conjugated anti-DIG Fab fragments (Roche catnr: 1093274). After rinsing in TNT and subsequently in NTM (100 mM Tris pH9.0 100 mM NaCl, 50 mM MgCl_2_), probe binding was visualized using nitro blue tetrazolium chloride and 5-bromo-4-chloro-3-indolyl-phosphate (Roche catnr: 1681451). Color development was stopped by rinsing in double-distilled water. The sections were dehydrated in a graded ethanol series, rinsed in xylene, and embedded in Entellan. Images were recorded using a Leica DFC320 camera mounted on an AxioPhot microscope (Zeiss).

### Immunofluorescent staining

Immunofluorescent staining was essentially performed as described. [24] In short, sections were deparaffinized, rehydrated in a graded ethanol series, boiled for 5 minutes in antigen unmasking solution (H3300, Vector), and incubated in PBS+1% Triton-×100 for 15 min. The following primary antibodies were used: anti-Troponin I (TnI) (MilliPore; catnr MB1691; 1∶500), anti-Wt1 (Santa Cruz; catnr SC-192; 1∶200), anti-PDGFRα (Santa Cruz; catnr SC-233; 1∶200), anti-CD34 (BD Bioscience; catnr 36105; 1∶200), anti-cKit (Southern Biotech; catnr 8380; 1∶200), anti-α-sma (Sigma; catnr A2522; 1∶1000), anti-BrdU (Becton Dickinson, catnr 347580; 1∶600), anti-P-Smad1/5/8 (Cell Signaling Technology, catnr 9511; 1∶200), anti-P-Erk1/2 (Cell Signaling Technology, catnr 4376; 1∶200), anti-PECAM (Santa Cruz; catnr SC-1506; 1∶100), and ß-Galactosidase (MP Biomedicals(Cappel), catnr 55976; 1∶500). To reduce background signal of the antiserum against ß-Galactosidase, the diluted antiserum was pre-incubated with mouse heart powder before application to the respective section. Mouse heart powder was prepared by homogenizing a wildtype adult mouse heart in PBS and precipitating the proteins with 4 volumes ice cold acetone. The precipitate was collected by centrifugation, the dry pellet was ground in a mortar and stored at −20°C.

Alexa488 or Alexa568 conjugated goat-anti-rabbit and goat-anti-mouse antibodies (Molecular Probes; 1∶250) were used as secondary antibodies. The signal was amplified using tyramide signal amplification (TSA NEL702, Perkin Elmer). Nuclei were visualized using Topro3 (Molecular Probes; 1∶500). Fluorescence was visualized using a Leica SPE confocal laser scanning microscope.

## Results

### Expression of embryonic epicardial genes in the adult healthy and infarcted heart

**Figure 1:**
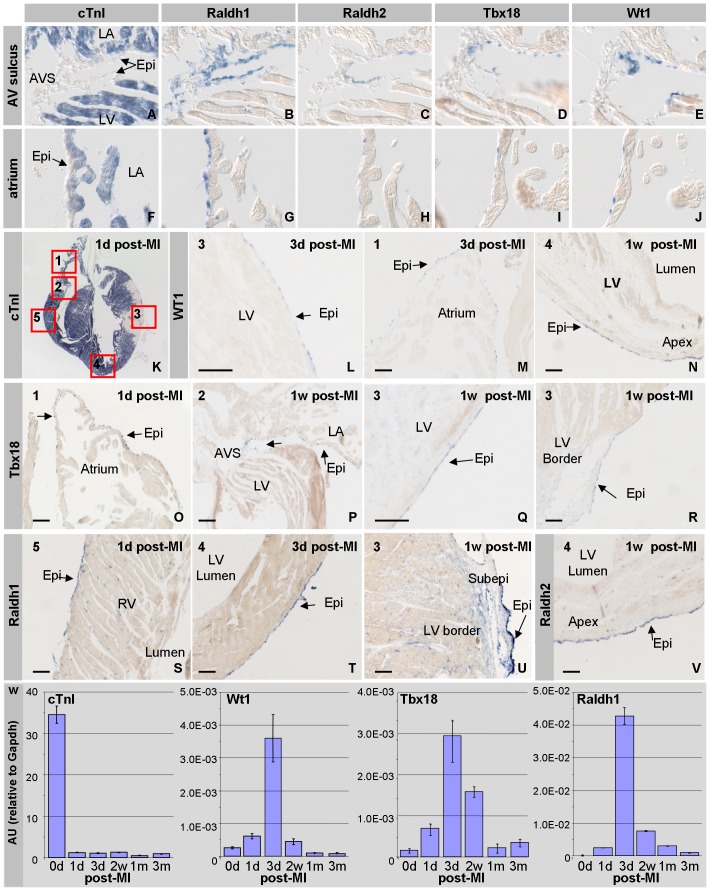
Epicardial activation in response to myocardial ischemia. Panels A–J show the pattern of expression of cTnI (A,F), Raldh1 (B,G), Raldh2 (C,H), Tbx18 (D,I) and Wt1 (E,J) mRNA in the normal healthy heart. Raldh1 (B), Raldh2 (C), Tbx18 (D) and Wt1 (E) are expressed in the epicardium and the adjacent subepicardial mesenchyme of the atrioventricular sulcus to various extends. Raldh1 (G) is expressed throughout the epicardium overlaying the atria. Raldh2 (H), Tbx18 (I) and Wt1 (J) are expressed in a small subset of the epicardial cells overlaying the atria. Panel K shows a representative four-chamber view of an adult heart one day post-MI; the absence of expression of cTnI mRNA demarcates the infarcted area. The boxes indicate the atrium (**1**), atrio-ventricular sulcus (**2)**, infarcted area of left ventricular free wall (**3**), the apex (**4**), and the right ventricular free wall (**5**). In the subsequent panels we have indicated this number at the left top site to indicate the position the picture was taken. Wt1 mRNA is expressed in the epicardium covering the ischemic region three days post-MI (L), to a level comparable to the one in the epicardium covering the atria (M). At one week post-MI, Wt1 is expressed in the epicardium covering the apex and left ventricle (N). Tbx18 (O–R) is expressed throughout the epicardium of the atria (O), within the atrioventricular sulcus (P), and the epicardium overlaying the infarct (Q) and it border zone (R). Raldh1 (S–U) is expressed throughout the epicardium covering the ventricles after a myocardial infarction (S,T), and in the subepicardial mesenchyme that is found in the wide subepicardial space overlaying the border zone of the infarction (U). Raldh2 (V) is expressed throughout the epicardium at one week post-MI. To validate these data we performed a qPCR analysis (W). As expected cTnI mRNA is down-regulated immediately after the induction of the infarction. Wt1, Tbx18 and Raldh1 mRNA become transiently up-regulated, being most highly expressed at 3d post-MI. Abbreviations: LA; left atrium, LV: left ventricle, RV: right ventricle, AVS: atrioventricular sulcus, Epi: epicardium, Subepi: subepicardium. The bar is 100 µm.

To gain insight into the response of the epicardium after a myocardial infarction we investigated the expression of markers for embryonic epicardium in the adult heart. During development, Tbx18, Wt1, Raldh1, and Raldh2 are expressed throughout the epicardial layer and in the adjacent mesenchyme, but all four gene products become down-regulated at the end of gestation [25]–[29]. Their expression during development is associated with the formation of subepicardial mesenchyme by epithelial to mesenchymal transition (EMT), as well as with myocardial growth and differentiation [30]. In the normal or diseased adult heart, their patterns of expression have not been described in detail [14]–[17]. In the adult heart Wt1, Tbx18, Raldh1, and Raldh2 mRNA were found to be expressed in the epicardium covering the atrioventricular sulcus (Figure 1 A–E) and the ventricular apex, but not in the epicardium covering the ventricles or in the ventricular wall. Raldh1 mRNA was also found in a subset of mesenchymal cells present in the atrioventriuclar sulcus (Figure 1B), whereas none of the other genes is found to be expressed in this mesenchyme (Figure 1A–E). Raldh1 mRNA was further found to be expressed in the epicardial layer covering the atria (Figure 1G). Although the other genes were also expressed in the epicardial lining of the atria, they were expressed at a low level and in a mosaic pattern (Figure 1H–J).

To investigate the changes in the spatio-temporal pattern of expression of these epicardial genes in relation to ischemia, we permanently ligated the LAD. To identify the ischemic region we hybridized an adjacent section with a cTnI probe. Already from the first day post-myocardial infarction (post-MI) the level of cTnI mRNA had dropped below detection in the ischemic myocardium (Figure 1K), allowing the identification of the site and extent of the infarct. At one day post-MI the expression of the embryonic epicardial markers had become more prominent in the regions expressing these markers in controls (eg Figure 1O, P, S), and from three days post-MI onwards their expression was detected in the epicardial cells covering the entire infarcted area as well as the border zone (Figure 1L, N, Q, R T, U). Raldh2 mRNA was, however, induced throughout the entire epicardial layer of the heart from the first day after the induction of the ischemia (Figure 1V). Concomitant with the appearance of mesenchymal cells in between the epicardium and myocardium of the border zone, these sub-epicardial mesenchymal cells were found to express the four embryonic epicardial genes as well, albeit at lower levels compared to the overlaying epicardium (Figure 1V). Although the patterns and levels of expression differed, the induction of their expression was transient, being confined to the first two weeks post-MI. At one and three months post-MI, the expression of the four embryonic epicardial genes was only found in the regions that also expressed these genes in healthy controls.

To further confirm the transient induction of the expression of these genes, RNA was isolated and the expression levels were determined by quantitative PCR. As expected the level of expression of cTnI mRNA was found to be approximately 100-fold lower in infarcted hearts from the first days after the induction of the ischemia up to the latest time point analyzed, being three months post-MI. The expression levels of Wt1, Tbx18 and Raldh1 mRNA were already found to be up-regulated at 1 day post-MI, showing the highest level of expression at three days post-MI and subsequently decreasing to return to levels similar to control at one and three months post-MI (Figure 1W).

### Analysis of the Wt1-lineage in response to ischemic injury

**Figure 2:**
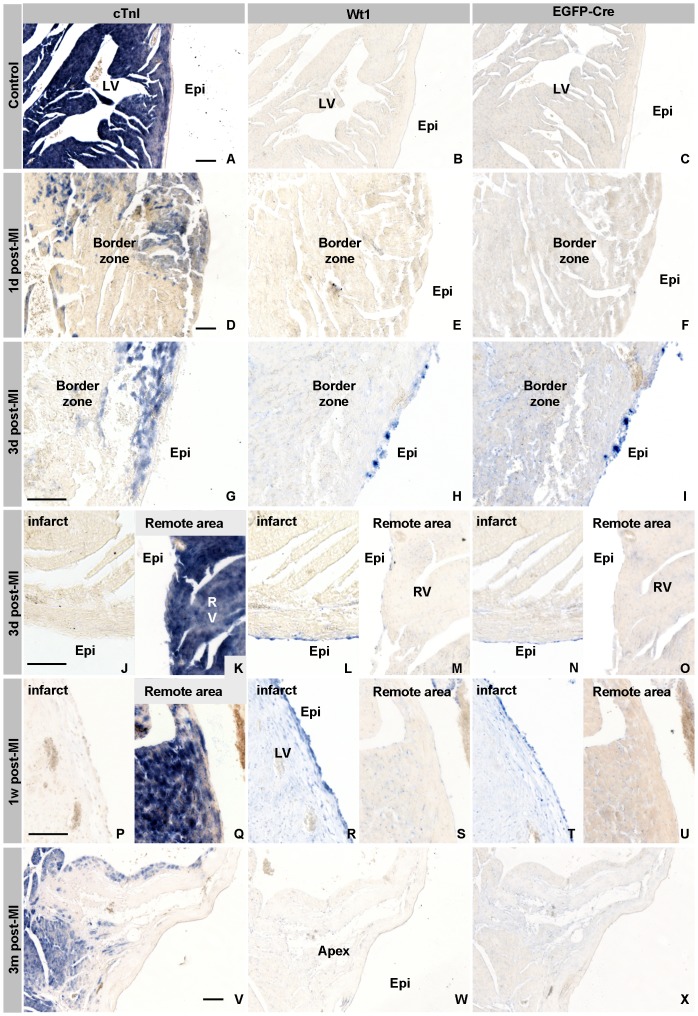
Comparison of the expression pattern of Wt1 and Cre in the healthy and infarcted heart. In the ventricles of control (A–C) Wt1^Cre^ mouse cTnI mRNA is expressed in all cardiomyocytes (A), whereas neither Wt1 (B) nor Cre (C) mRNA are detected in the ventricles. One day after LAD ligation (post-MI) (D–F), the expression level of cTnI mRNA has dropped below detection in the infarcted zone and is expressed at a lower level in the cardiomyocytes of the border zone than in remote healthy myocardium (D). At one day post-MI both Wt1 and Cre (E & F) are not detected. Three days post-MI (G–O), the expression of both Wt1 (H L) and Cre (I, N) are found in the epicardial cells covering the border of the infarcted area (G) and the infarct proper (J), but not covering remote, healthy myocardium (K, M & O). One week post-MI (P–U), the pattern of expression of Wt1 (R, S) and Cre (T, U) is essentially the same as at three days post-MI. Three months post-MI (U–W), Wt1 (W) and Cre (X) are, like in the control heart, not longer detectable. Abbreviations: LV: left ventricle, RV: right ventricle, Epi: epicardium. The bar is 100 µm.

Patterns of gene expression do not allow an assessment of the fate of the epicardial cells after the myocardial infarction. In order to assess the fate of the epicardial cells, we needed to permanently label the epicardial cells. We have recently described a Wt1^Cre^ mouse line, which permanently labels the proepicardium, the epicardium and its derivatives, and in a limited number of small clusters of cardiomyocytes within the ventricular septum [19]. These observations suggest that this mouse line might be a useful tool to study the epicardial lineage after a myocardial infarction [31], [32]. As the Cre/loxP system relies on the similarity of the spatio-temporal pattern of expression of Wt1 and Cre, we compared the expression patterns of Wt1 and Cre mRNA in the healthy and infarcted heart. Like Wt1 mRNA, Cre mRNA was not detected in the epicardium overlaying the left and right ventricular free wall or within the adjacent myocardial wall (Figure 2A–C). This expression pattern was not found to have changed 1 day post-MI (Figure 2D–F). At three days post-MI both Wt1 and Cre mRNA expression were detected in the epicardium overlaying the border zone of the infarct (Figure 2G–I) as well as the infarction itself, albeit at lower levels (Figure 2J, L and N), and was absent from epicardium located remotely of the infarct (Figure 2K, M, and O). At one week post-MI Wt1 and Cre mRNA expression had become more prominent in the area of the infarct, being expressed in the epicardium and immediately adjacent mesenchyme (Figure 2P–U). At two weeks post-MI the expression levels of Wt1 and Cre mRNA had decreased (not shown), and were no longer detectable in the infarcted region at 1 and 3 months post-MI (Figure 1V–X). Although the findings with respect to Wt1 mRNA are in line with a previous report [33], we did not observe expression of Wt1 mRNA in the coronaries in the infarcted region, even if the sections were over-stained (data not shown).

Taken together, these observations show that the spatio-temporal pattern of expression of Cre mRNA parallels that of Wt1 mRNA, indicating that this Wt1^Cre^ mouse line, concerning this aspect of the Cre/loxP analysis, can be used to trace the fate of Wt1 expressing cells in the region of the infarction.

**Figure 3:**
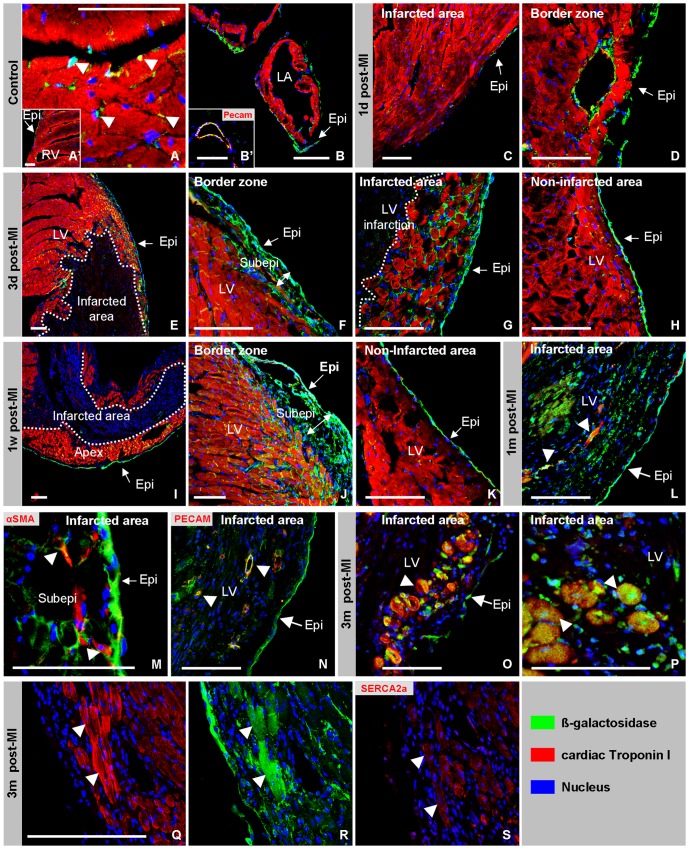
Post ischemic epicardial lineage analysis. In healthy *Wt1^Cre^ X R26R* hearts ß-Gal is detected in the entire epicardium covering the ventricles, atria and atrioventricular junction (A, B). βGal is also detected in cells of the coronaries and in inter-myocardial cells (A, A′). One day post-MI, no epicardial ß-Gal-expressing cells were observed to cover the infarcted area (C). In the epicardium and coronaries of the border zone ß-Gal was still present (D). Three days post-MI, ß-Gal-expressing epicardial cells covered the infarcted area (E). In the border zone a broadened (double arrow) subepicardial ß-Gal-expressing layer of cells appears (F,G). In the uninjured areas of the same heart ß-Gal expression was comparable to the control situation (H). The dotted line highlights the border between healthy and infarcted myocardium. One week post-MI the subepicardial layer has further broadened (double arrow, cf F & J) and the number of subepicardial ß-Gal-expressing cells has increased (J). One month post-MI the ß-Gal-expressing cells are present in the infarcted area and the epicardium covering the infarcted area (L–N). A subset of the ß-Gal-positive cells co-express cTnI (L), α-SMA (M) or PECAM (N). Three months post-MI the number of ß-Gal- and cTnI-co-expressing cells has increased and these cells are found throughout the infarcted area (O & P). Panel Q–S show consecutive sections showing the expression of cTnI, ß-Gal and SERCA2a. The arrow shows cardiomyocytes that are positive for all three markers. Abbreviations: LA: left atrium, LV: left ventricle, RV: right ventricle, Epi: epicardium, Subepi: subepicardium. The bar is 100 µm.

Since this Wt1^Cre^ mouse line encodes a constitutive, rather than an inducible, Cre, the lineage analysis will not only show the effects that are the result of the induced myocardial infarction, but also the developmental history. To visualize the result of the developmental expression of Wt1 in the adult heart, we crossed male Wt1^Cre^ with female R26R reporter mice and used immunofluorescent staining to identify the ß-Galactosidase (ß-Gal) expressing cells. As expected. the entire epicardium and adjacent mesenchyme, a subset of the endothelial cells of the coronaries and intermyocardial fibroblasts were found to express ß-Gal in the adult hearts (Figure 3A,B). We did not identify ß-Gal-expressing cardiomyocytes in the ventricular free wall, which is in line with our developmental analysis [19] but in contrast to the Wt1 Cre knock-in lines [31], [34].

Next we established the ß-Gal-expression pattern at various time points after permanent ligation of the LAD. To identify the ischemic area, we co-stained the sections for cTnI. The cTnI expression level is decreased at 1 day post-MI and below detection from three days post-MI onward in the infarcted region (Figure 3). At 1 day post-MI the ß-Gal-expressing epicardium overlying the border zone of the infarcted area showed interruptions, and was largely absent from the infarcted area (Figure 3C–D). Classic histological staining of neighboring sections confirmed that the epicardial layer covering the ischemic area had disintegrated (data not shown), which is in line with previously published data [14]. Three days post-MI the infarcted area was found to be covered with a contiguous ß-Gal-expressing epicardial layer (figure 3E–H). These observations suggest that the epicardial layer overlaying the ischemic area is lost and regenerated within three days after the induction of ischemia. At three days post MI not only the epicardium was regenerated but also the space between the myocardium and epicardium covering the border zone of the infarction had widened and had become populated with ß-Gal-expressing mesenchymal cells (Figure 2F). In areas remote of the infarction and in controls, there was virtually no space between the epicardium and the ventricular myocardium (compare Figure 3F, J with 3H, K). At one week post-MI the space between the ß-Gal-positive epicardium and the myocardium had further widened and was no longer restricted to the border zone but included the entire infarct and was extensively populated with ß-Gal-expressing mesenchymal cells (Figure 3I–K). At one month post-MI, the wide ß-Gal-positive mesenchymal layer was still present in the border zone, but had disappeared from the infarcted area. ß-Gal-positive cells were now found throughout the infarcted area, suggesting that the subepicardial mesenchyme had invaded and replaced dead cardiomyocytes in the infarct (Figure 3L). This became even more evident at three months post-MI (data not shown). Although ß-Gal-positive cells contribute to the infarcted region not all cells are positive, increasing from 14±2% of the cells at one week post-MI to 38±5% of the non-myocardial cells within the infacted area, excluding the epicardium and subepicardial mesenchyme. These findings indicate that the non-myocytes in the infacted area are derived from other ancestries as well, being the endocardium [35], [36] and the bone marrow [37].

To gain insight into the phenotype of the ß-Gal-positive cells in the infarcted region triple immunofluorescent staining was performed. Based on this analysis the ß-Gal-expressing cells could be subdivided in four groups: (*i*) Cells co-expressing α-Smooth Muscle Actin (α-SMA) (Figure 3M), that were found throughout the infarcted area and adjacent to blood vessels. (*ii*) Cells co-expressing PECAM (CD31) that were located within coronary vessels (Figure 3N). (*iii*) Cells co-expressing cTnI, qualifying them as cardiomyocytes (Figure 3O–P). (*iv*) Cells co-expressing none of the three above markers, qualifying them as interstitial cells (Figure 3M–P). It should be noted that the cardiomyocytes comprise small islands of small rounded cells that most often do not show the typical architecture of adult cardiomyocytes. Due to their non-random distribution it is impossible to reliably quantify them, nevertheless we estimate that this population comprises a few hundred cells per infarct. With respect to this observation it is relevant to note that cTnI and ß-Gal double-positive cells (1) were only found at 1 and 3 months post-MI, (2) were never found in the left ventricular free wall outside the infarcted area, and (3) were never found in the ventricular free wall of controls. (4) Moreover, these cells were besides positive for cTnI also positive for other cardiomyocytes markers, like sarcoplasmic reticulum calcium ATPase 2a (SERCA2a).

### Proliferation of Epicardial and mesenchymal cells

**Figure 4:**
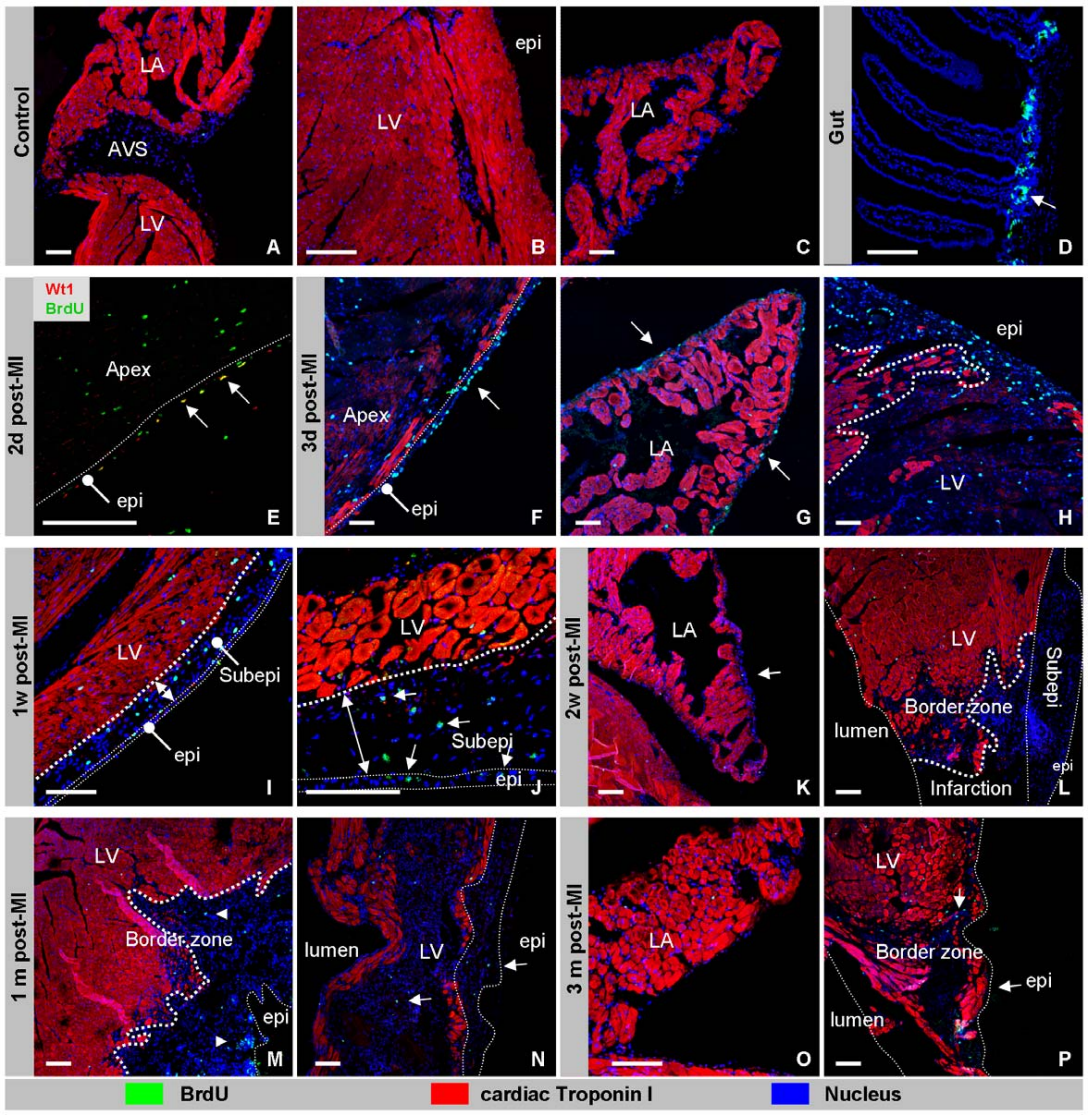
Proliferation of epicardial and subepicardial cells upon myocardial ischemia. In control hearts BrdU was not detected in the epicardium covering the atrioventricular junction (A), left ventricle (B) and atrium (C). As a positive control, BrdU incorporation was confirmed in the crypts of the gut in each mouse (D). At two days post-MI BrdU was detected in Wt1-positive epicardial cells (arrows) and some Wt1-negative cells throughout the myocardium of the apex (E). The thin dotted line highlights the border between the epicardium and underlaying myocardium. At three days post-MI BrdU-positive cells are found within in the epicardium covering the infarcted area (F), atria (G), and in mesenchymal cells in the border zone of the infarct (H). At one week post-MI BrdU was detected in cells located in the subepicardial space (double arrow) covering the infarcted area (I, J). At two weeks post-MI, BrdU was detected in a few individual subepicardial cells of the atria (arrow) (K) and of the border zone of the infarct (L). At one and three months post-MI hardly any BrdU incorporation was detected in the heart (M–P). Abbreviations: LA; left atrium, LV: left ventricle, RV: right ventricle, AVS; atrioventricular junction, Epi:epicardium, Subepi: subepicardial mesenchyme. The bar is 100 µm.

The observation that the epicardium overlaying the infarcted region is regenerated within the first three days after the infarct and the formation of a thick layer of subepicardial mesenchyme over the infarcted area, suggests that the healthy epicardium from the border zone grows over the infarct and that the newly formed epicardium proliferates and undergoes EMT to provide the subepicardial mesenchyme. To investigate these possibilities, mice were labeled with BrdU for 4 hours before sacrifice. As a positive control gut tissue was isolated and analyzed in parallel (Figure 4D). In control hearts, as expected [38], only an incidental non-myocardial cells was found to have incorporated BrdU (Figure 4A–C). One day post-MI, BrdU-incorporation was observed in the epicardial cells overlying the border zone of the infarct (Figure 4E) and in a low number of epicardial cells covering the atria (data not shown). We also observed BrdU incorporation in endocardial cells throughout the entire heart. During the following two weeks post-MI, BrdU incorporation was detected in the epicardium and mesenchymal cells overlying the infarct, but not in epicardial cells covering uninjured remote ventricular myocardium (Figure 4F–L). At 1 and 3 months post-MI, BrdU-incorporation was largely similar to controls, except for an occasional individual mesenchymal cell in the border zone of the infarct (Figure 4M–P). These observations suggest that the lost epicardium is regenerated from healthy epicardium covering the border zone. Secondly, the finding that the epicardium overlaying the infarct and its adjacent mesenchyme is not only ß-Gal-positive but also has incorporated BrdU, further underscores the idea that the subepicardial mesenchyme is *de novo* formed by EMT from the overlaying epicardium.

### Subepicardial mesenchyme formation by EMT

**Figure 5:**
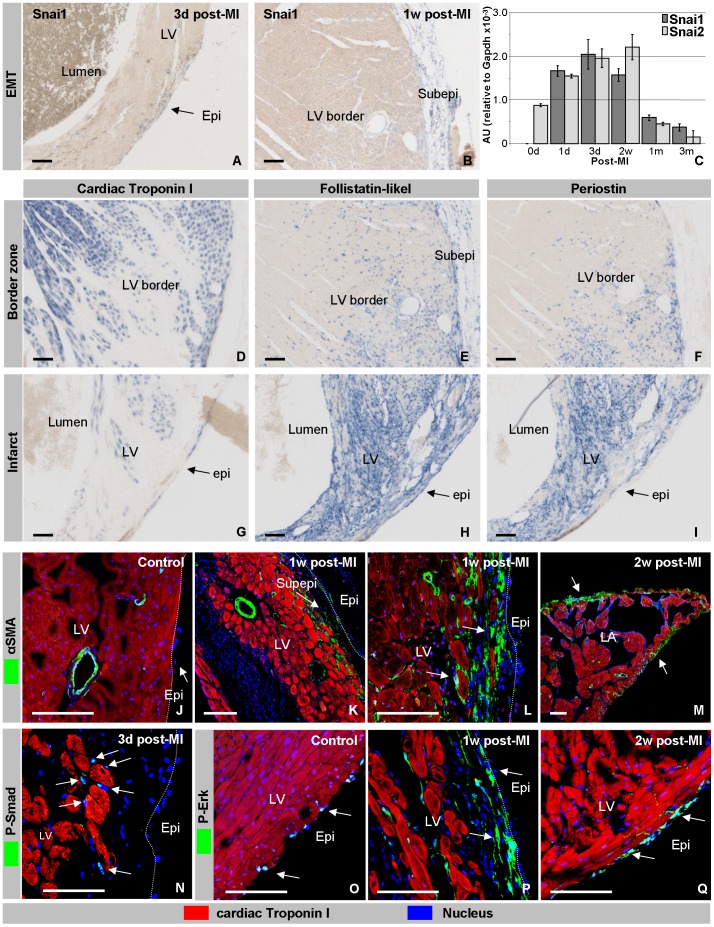
Post-ischemic mesenchyme formation. Snai1 mRNA is present from three days onwards in the subepicardial mesenchyme of the apex and LV covering the infarct (A), and border zone (B). qPCR analysis (C) showed that Snai1 is already induced at one day post-MI and remains high up to two weeks post-MI, after which it gradually returns to control levels. Its close relative Snai2 shows a similar activation pattern as Snai1, with the exception that Snai2 is already detected in the healthy control heart. Within the border zone (D) and infarcted area (E) the matricellular proteins Follistatin-like1 (E,H) and Periostin (F,I) are expressed at high levels in the non-myocardial cells (cf D,G). In control hearts, α-SMA is present in the smooth muscle layer of the coronaries (J). At one and two weeks post-MI α-SMA is detected in the subepicardial mesenchyme and in mesenchymal cells located in the myocardium adjacent to the epicardium overlaying the infarct (K,L). At one and two weeks post-MI α-SMA-postive subepicardial mesenchyme is also found in the atria (M). P-Smad is only found in a small minority of subepicardial mesenchymal cells (arrow) directly flanking myocardial cells and adjacent to the infarct (N). Whereas P-Erk is detected in the nucleus of a subset of epicardial cells in controls and after an infarction, as well as in the cytoplasm and nucleus of an extensive subset of the subepicardial mesenchyme (O–Q). The bar is 100 µm.

Both the proliferation and lineage analysis point to *de novo* mesenchyme formation from the overlaying epicardium by EMT. To further substantiate this idea the pattern of expression of Snai1 and α-SMA, markers of EMT during embryogenesis [39], were determined in the region of the infarct. Concomitant with the appearance of mesenchymal cells adjacent to the epicardium covering the border zone and the infarcted area, expression of Snai1 (Figure 5A–C) and α-SMA (Figure 5J–M) were found in both the epicardial and mesenchymal cells. In areas remote of the infarct and in controls expression of Snai1 was not observed. At two weeks post-MI, the number of cells expressing these markers had decreased and at three months post-MI only a few scattered mesenchymal cells were found in the border zone. Because Snai1 is closely related to Snai2, we prepared primers to amplify the two genes separately. The qPCR analysis showed that Snai1 mRNA is not detectable in control hearts, was induced at 1 day post MI, and starts to decrease after twee weeks post-MI, to return to almost undetectable levels at three months post-MI (Figure 5C). Its relative Snai2 was detected in control hearts, and follows a similar temporal expression profile as Snai1. To exclude that the primers were not sufficiently specific, the PCR products were cloned and sequenced. Taken together these findings further underscore our interpretation that at least a subset, if not all, mesenchymal cells found between the epicardial layer and the infarcted myocardium are formed by EMT from the overlaying epicardium.

Another group of proteins regulating EMT are matricellular proteins [40], [41]. These proteins function by their ability to interact with multiple cell surface receptors, as well as by binding directly to structural or scaffold proteins. The expression of this family of proteins is most prominent during development but has also been shown to play an important role in the response to injury [40]. We investigated the expression of two members of this family, Follistatin-like-1 (Figure 5E,H) and Periostin (Figure 5F,I), that both have been shown to be involved in mesenchyme differentiation in the developing heart. Fstl1 and Periostin mRNA were both present in the infarcted zone, with Fstl1 being more extensively expressed than Periostin (Figure 5D–I). Both mRNAs were most abundantly expressed at one week post-MI, after which their expression gradually decreased to very low, but still detectable levels, at two weeks and becoming undetectable from one month post-MI onward.

### Regulators of mesenchyme differentiation

Supplementing medium conditioned by EPDCs to mice, was found to reduce the size of the infarct and improve cardiac function [32]. Recently, expression of Wnt1 was found to be induced in the epicardium and adjacent infarct tissue, eliciting a pro-fibrotic response [42]. During development a progenitor cell population located upstream of the inflow of the heart contributes cells to the myocardium and epicardium. Inhibiting Wnt signaling during development by applying recombinant Wif1 to the developing embryo was found to increase this progenitor pool [43]. The cooperative interaction of Smad-mediated BMP and Erk-mediated FGF-signaling subsequently directs the differentiation of these progenitors into either the myocardial or epicardial lineages [11], [44]. Since the lineage analysis had identified a small population of ß-Gal-positive cardiomyocytes, we determined whether P-Smad and P-Erk are expressed after a myocardial infarction. P-Smad was found to be expressed at low levels in mesenchymal cells flanking the cardiomyocytes of the border zone and in scattered cells throughout the infarcted area (Figure 5N). P-Erk, on the other hand, was observed in a small portion of epicardial cells in control hearts. At three days and one week post-MI P-Erk was observed at high levels in the mesenchyme adjacent to the epicardium overlaying the infarcted area (Figure 5O–Q). At one month post-MI, the mesenchymal cells expressing P-Erk were only found in mesenchymal cells in the border zone of the infarct. At three months post-MI the expression pattern was found to be similar to controls. P-Smad was found to be expressed at low levels in mesenchymal cells flanking the cardiomyocytes of the border zone and in scattered cells throughout the infarcted area (Figure 5N). Taken together the pattern of expression of P-Erk suggests a role in epicardial EMT, and that of P-Smad a role in the differentiation of cardiomyocytes in the infarcted.

### Expression of stem cell markers in adult healthy and infarcted hearts

**Figure 6:**
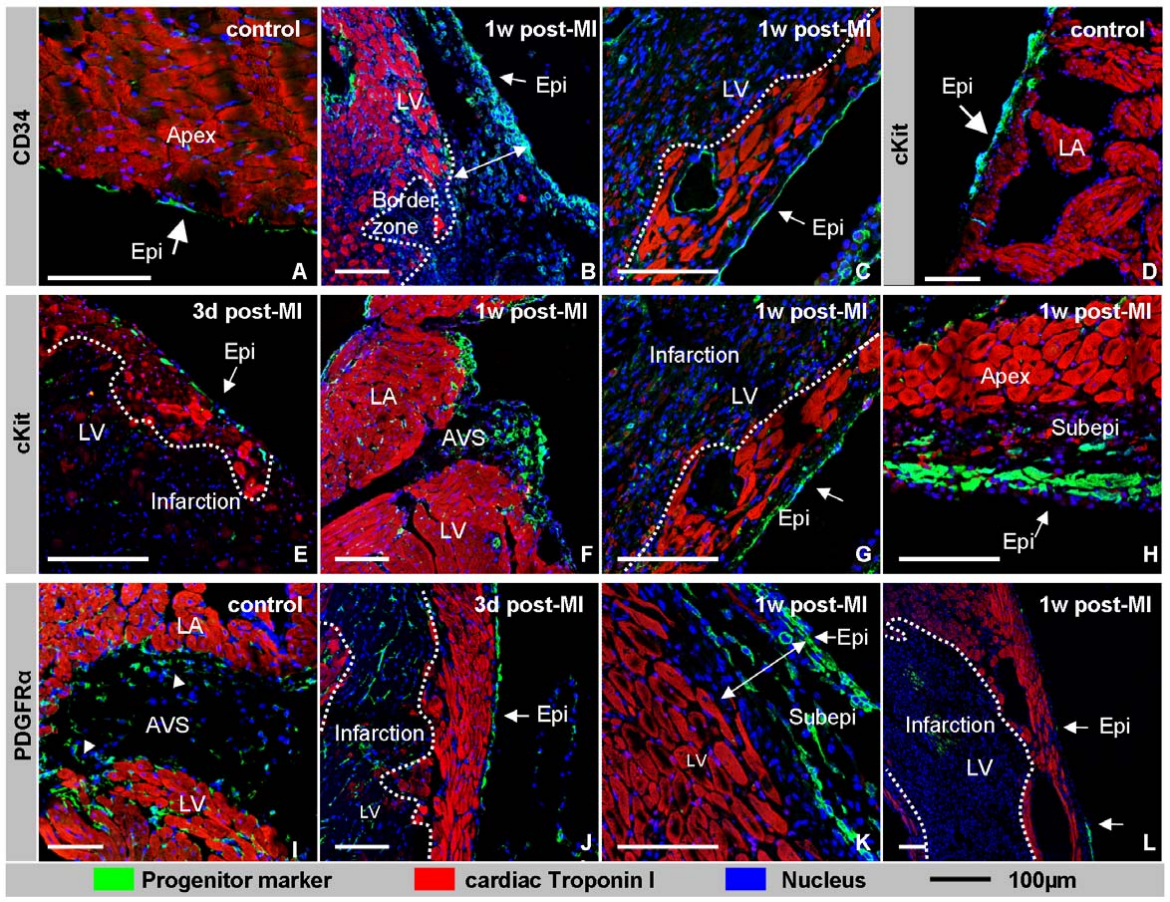
Expression of stem cell markers. The different stem cell markers, CD34 (A–C), cKit (D–H) and PDGFRα (I–L) are shown in green, cTnI in red, and the nuclei in blue. In the healthy heart CD34-positive cells are found to be scattered through the epicardium covering the atria (A). After the induction of the ischemia CD34 is up-regulated and becomes highly expressed in the epicardium covering the infarct and its border zone (B,C), as well as in mesenchymal cells in the subepicardial space in the border zone (B). cKit is expressed in the epicardium and individual adjacent mesenchymal cells of the atria in control hearts (D). cKit becomes evident in scattered cells of the epicardium overlaying the border zone of the infarct at 3 days post-MI (E). At one week post-MI cKit is expressed throughout the epicardium overlaying the infarct and its border zone, as well as in a subset of immediately adjacent mesenchymal cells (G, H). Moreover, at this time point cKit is also expressed in the epicardium and a subset of adjacent subepicardial mesenchymal cells in the atrioventricular sulcus (F). In control heart PDGFRα is expressed in a small portion of the mesenchyme of the atrioventricular sulcus (I). At three days post-MI (J) and one week post-MI (L) PDGFRα is expressed in the epicardium overlaying the infarction. In the border zone PDGFRα is also expressed in mesenchymal cells within the subepicardial space (L). The dotted line highlights the border between infarcted tissue and healthy myocardium. The double arrow highlights the width of the subepicardial space. Abbreviations: LA: left atrium, LV: left ventricle, Epi: epicardium, AVS: atrioventricular sulcus. The bar is 100 µm.

Upon amputation of the cardiac apex in zebrafish, the epicardium is activated, and myocardial cells start to proliferate [9], [10], [45]. Like the zebrafish heart, the neonatal mouse heart is able to respond to injury [7], whereas in the adult mouse heart proliferation of cardiomyocytes after a myocardial infarction was not observed (Figure 4) [38]. Also in this study we did not observe any BrdU incorporation in adult cardiomyocytes, suggesting that it is very unlikely that the βGal-expressing cardiomyocytes are derived from existing cardiomyocytes by proliferation. As an alternative to *de novo* differentiation of epicardium-derived cells, the cardiomyocytes might originate from differentiation of stem cells that express or have expressed Wt1. The latter option seems to be a likely option because Wt1 has been shown to be important for the formation of progenitor cells in the epicardium during development [39], and, secondly, the adult epicardium has been suggested to be a source of stem cells [46]. To evaluate the changes in the potential progenitor population, we determined the pattern of expression of Platelet Derived Growth Factor α (PDGFRα), CD34 and cKit by immunofluorescence (Figure 6). Unfortunately, it is technically impossible to perform reliable triple immunufluorescent staining for ß-Gal and these stem cell markers because the primary antibodies are raised in the same species. In the healthy heart PDGFRα, CD34 and cKit were detected in the epicardium covering the atria, atrioventricular sulcus and apex. PDGFRα and CD34 were also detected in a subset of mesenchymal cells in the atrioventricular sulcus (Figure 6A, D, I). This finding is in line with the identified stem cell niches [47] and with the sites of expression of Wt1. At three days post-MI, cKit, but not PDGFRα and CD34, expression was observed in a portion of the epicardial cells that cover the border zone of the infarct (Figure 6E). At one and two weeks post-MI expression of all three markers was found in a subset of the mesenchymal cells adjacent to the epicardium overlaying the infarct (Figure 6B, C, F–H, K–L). From one month onward the expression of the stem cell markers was no longer detected in the area of the infarct anymore, but only in the regions found in controls (data not shown). Since the spatio-temporal pattern of expression of the stem cell markers and the cardiomyocytes markers do not overlap in the region of the infarct, this analysis does not imply a direct association. Since our analysis has shown that a large part, if not all, subepicardial cells overlaying the infarct are formed *de novo* by EMT from overlaying epicardium, one might speculate that after an infarction the epicardium-derived stem cell population expands and provides the precursors of the various Wt1-derived populations (Figure 3 M–P). Nevertheless, the origin of the Wt1-lineage positive cardiomyocytes remains enigmatic.

## Discussion

Traditionally, the epicardium has been regarded as a mesothelial sheath protecting the underlying myocardium and producing the pericardial fluid facilitating cardiac contraction. However, recent research has revealed that the epicardium is far from being a static anatomical structure, but plays a pivotal role in homeostasis and regeneration of the adult heart. Using the epicardium as a source of cells in novel regenerative strategies might, therefore, be an attractive option. A prerequisite for developing such novel strategies is the understanding of the cellular and molecular changes in the epicardium during regeneration.

**Figure 7:**
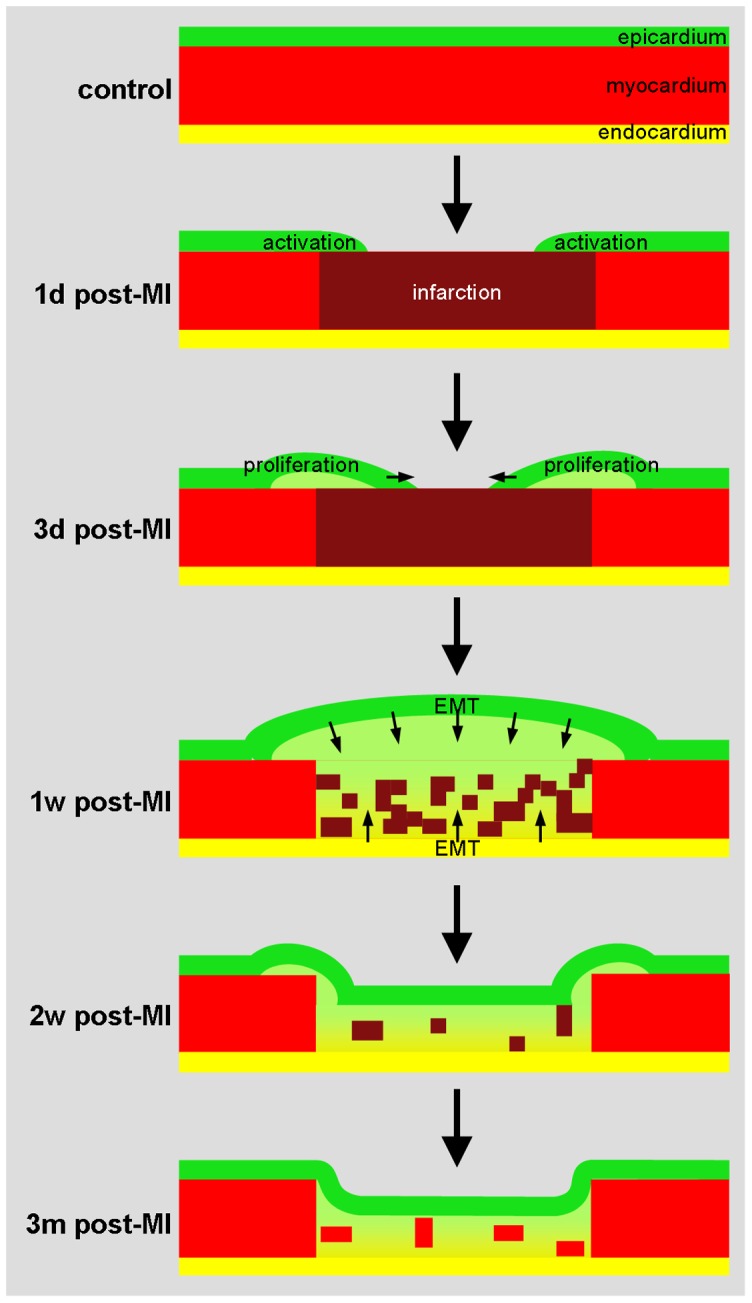
Schematic overview of morphological and molecular changes upon myocardial ischemia.

The results obtained by using in situ hybridization, immunofluorescence and classic histological analyses are summarized in a schematics (Figure 7). At one day post-MI most of the epicardium overlaying the ischemic region is disrupted. The epicardial layer immediately adjacent to the ischemic zone, i.e. the border zone, starts to express embryonic epicardial genes, starts to proliferate, and the space between the epicardium and myocardium widens. Within the epicardium of the border zone the expression of Wt1 mRNA and protein, as well as Cre is induced. At three days post-MI the ischemic myocardium is again covered with an epicardial layer that is separated from the underlying myocardium by a wide gap. This epicardium is expressing Wt1, Cre and βGal. Taken together these observations suggest that the new epicardial layer overlaying the infarct is formed from the epicardium overlaying the border zone. In line with this interpretation the epicardial cells overlaying the border zone express genes that are also expressed during formation of epicardium during development.

The initially a-cellular gap between the newly formed epicardium and infarct becomes populated by mesenchymal cells. Tracing the Wt1-lineage showed that these mesenchymal cells are βGal-positive. This observation indicates that the mesenchymal cells are descendants of cells that express or have expressed Wt1. Therefore, these mesenchymal cells are derived from the overlaying epicardium or have invaded from a remote location. The following observation suggests that the mesenchymal cells are derived from the overlaying epicardium. The BrdU labeling experiments showed incorporation of this proliferation marker in cells of the epicardium overlaying the infarct and in adjacent mesenchymal cells. Though one could conclude that both cell populations are proliferating, one could also regard the incorporated label as a short term lineage tracer, because the mice were exposed to BrdU 4 hours prior to sacrifice. As such the finding of BrdU labeling in both the epicardium and subepicardial mesenchyme mesenchymal cells suggests that the mesenchymal cells are derived from the overlaying epicardium by a process of EMT. This conclusion is underscored by the observed expression of the EMT markers Snai1 and αSMA. Nevertheless, we cannot exclude that descendants of Wt1-expressing cells have invaded the infarct area from remote sites, as in the healthy heart Wt1-expressing cells have been identified in stem cells niches in the atrioventricular sulcus and apex of the ventricle and, secondly, that Wt1 has been shown to be important for the formation of progenitor cells in the epicardium during development [39].

After the induction of the ischemia the gap between the epicardium and infarcted myocardium initially widens forming an extensive mesenchymal layer. The mesenchymal cells within this layer are virtually all derived from the Wt1-lineage, as they express Wt1, Cre and/or βGal. At this point it is of relevance to note that the Wt1 and Cre are not expressed in this region of the healthy controls, further underscoring the reactivation of the epicardium in the infarcted region. Subsequently, at one and three months post-MI, the mesenchymal layer starts to thin, as the mesenchymal cells start to occupy the space of dead cardiomyocytes. Interesting, in the forming infarct scar more than one third of the cells are positive for βGal, indicating that not only cells of the Wt1-lineage contribute to the scar tissue. This finding is in line with analyses showing a contribution of endocardium-derived cells [35], [36], bone marrow-derived cells [37], as well as a small contribution of cardiomyocytes [48] to the scar.

During development epicardium-derived cells contribute to the intermyocardial fibroblast population and the coronary vessels [39]. To evaluate whether after an infarction the epicardium-derived cells, i.e. the Wt1-lineage positive cells, also contribute to these populations a triple immunofluorescent analysis was performed. This analysis showed that a small portion of the Wt1-lineage positive cells also expressed CD31, qualifying them as endothelial cells. The identification of Wt1-lineage positive coronary endothelial cells was unexpected based on a mouse study [49], but expected based on studies in chicken [50], [51]. Our Wt1-lineage analysis together with the analysis of the pattern of expression of Wt1 mRNA and protein suggests that the coronary endothelial cells are *de novo* formed. Alternatively, the CD31-expressing Wt1-lineage positive cells are the result of outgrowth of coronaries from the border zone into the infarcted area. Although we were not able to identify Wt1 mRNA or protein expression in the border zone of the infarct, it has been reported that after an infarct Wt1 expression is induced in coronary endothelium in the border zone [33].

The identification of a small number of Wt1-lineage positive cardiomyocytes from one month post-MI onward was unexpected. Epicardial-to-myocardial differentiation is a contentious topic based on mouse and chicken studies [18], [31], [32], [34], [52]. Our first concern was the specificity of our staining. However, the spatio-temporal pattern of the cardiomyocytes and the fact that they are positive for different markers does underscore the specificity of the staining. Another issue is the fact that only a very limited number of cardiomyocytes is found. Because the labeling of cells in the Cre/lox system is the result of a stochastic process, low numbers of positive cells might be the result of random recombination and thus as such illegitimate labeling. However, this does not seem to be an issue as Wt1-lineage positive myocytes are only observed in the infarct at one and three months post-MI and never in control hearts or in infarcts prior to one month post-MI. Moreover, if the labeling was due to illegitimate recombination then one would expect a random distribution throughout the infarct or even the entire heart, which is not the case.

Epicardium-derived cardiomyocytes have been reported in zebra fish upon amputation of the apex [10] and, in a recent study in mice, after a myocardial infarction [18]. Nevertheless, the origin of these cardiomyocytes is largely unknown. Although our Wt1-lineage analysis suggests that the myocytes are direct daughters of the *de novo* formed epicardial layer covering the infarcted zone, other options are possible. These myocytes might be derived from the stem cell population that has expressed Wt1 early during its life-span and resides in the heart. Alternatively, these cardiomyocytes might be the result of fusion of lineage positive mesenchymal cells with existing cardiomyocytes, a phenomenon also occurring during stem cell transplantation [53]. The observation that most of the myocytes do not have the characteristic adult cardiomyocyte shape and contain only one nucleus, renders this latter option unlikely. Nevertheless, experiments need to be performed to falsify this option. Taken together, these observations point to a Wt1-derived population of cells, that is capable of differentiating into the myocardial lineage after a myocardial infarction. When the efficiency of this process could be enhanced, this population forms an attractive source of new cardiomyocytes in a regenerative approach [6].

### Pro-fibrotic mesenchymal signaling inhibits cardiomyocyte formation

The limited myocardial regenerative capacity of the mouse heart might be hard wired or the net outcome of the integration of various signaling pathways. During early development the extent of the Wt1-expressing progenitor population at the inflow of the heart is regulated by Wnt signaling [43], and the subsequent differentiation into the epicardial or myocardial lineages by the cooperative interaction of Erk-mediated FGF-signaling and Smad-mediated BMP-signaling [11]. In the developing epicardium BMP-mediated myocardial differentiation is inhibited by Erk-mediated phosphorylation of Smad, rendering it incapable of accumulating in the nucleus and conveying the BMP signal [11]. With further development, the epicardium secretes growth factors, like FGF and BMP that stimulate proliferation of the underlying myocardium [30]. Also the adult epicardium was found to secrete growth factors that modulate the extent of the injury after a myocardial infarction [32], [42]. Immunofluorescent analysis of the infarcted area identified abundant P-Erk expression in the mesenchyme, as well as very limited and low levels of expression of P-Smad. These data suggest that in the infarcted region, like at the inflow of the developing heart, Erk-mediated FGF-signaling stimulates the formation of non-myocardial cells and inhibits cardiomyocyte differentiation via a cooperative interaction [54]. This idea is further supported by the observation that Fstl1, a natural BMP inhibitor [55]–[57] is expressed at high levels in the infarct, and as such inhibits cardiomyocyte differentiation. At first glance contradictory to our idea, ectopic expression of Fstl1 [58], [59] or inhibition of FGF-signaling [60] reduces infarct size. This reduction in infarct size is, however, not due to formation of new cardiomyocytes, but the result of induced/enhanced revascularization, enhancing survival of cardiomyocytes. Also the matricellular protein, Periostin, which directs differentiation of mesenchymal cells into fibroblasts [61], [62] and upon disruption results in reduced infarct size [63], was found to be expressed at high levels in the infarct.

### General conclusion

Taken together, this analysis shows that a regenerative response, similar to the response observed in fish, is initiated in the mouse upon MI, suggesting that an evolutionary conserved mechanism persists in the mammalian heart. However, unlike in fish, the regenerative response in mammalian heart is abrogated, resulting in the formation of only a limited number of new cardiomyocytes. Our findings provide basic knowledge that will be useful for the development of new strategies to enhance the endogenous regenerative capacity of the epicardial lineage.
